# Strain in Metal Halide Perovskite Thin Films - Interfacial Mechanical Coupling

**DOI:** 10.1021/acsenergylett.6c00526

**Published:** 2026-05-14

**Authors:** Zihan Zhang, Collin A. Sindt, Gabriel R. McAndrews, Samantha C. Kaczaral, Wenhan Ou, Nicholas J. Weadock, Michael D. McGehee, Michael F. Toney

**Affiliations:** † Department of Physics, 1877 University of Colorado Boulder, Boulder, Colorado 80309, United States; ‡ Department of Chemical and Biological Engineering, University of Colorado Boulder, Boulder, Colorado 80309, United States; § Materials Science and Engineering Program, University of Colorado Boulder, Boulder, Colorado 80309, United States; ∥ Renewable and Sustainable Energy Institute (RASEI), University of Colorado Boulder, Boulder, Colorado 80309, United States

## Abstract

Hybrid organic–inorganic metal halide perovskites (MHPs) are promising semiconductors for photovoltaics and optoelectronics, but their commercial viability is limited by instability, particularly strain induced by mismatch in coefficients of thermal expansion (CTE) between the perovskite film and substrate. Here, we investigate strain development and relaxation in MHP thin films using in situ bending and Grazing Incidence Wide-Angle X-ray Scattering (GIWAXS). We quantify the film–substrate interfacial mechanical coupling and identify interfacial slippage beyond a critical strain (∼0.4%), with Br-2PACz exhibiting comparatively stronger interfacial mechanical coupling among common interface modifiers. Time-resolved GIWAXS reveals reversible macrostrain during thermal cycling driven by CTE mismatch. Leveraging this behavior, we introduce a prestrain process that induces persistent compressive strain after cooling, with partial relaxation over time. These results provide insight into interfacial mechanical coupling and strain dynamics, offering a framework for strain engineering in perovskite devices.

## Graphical abstract

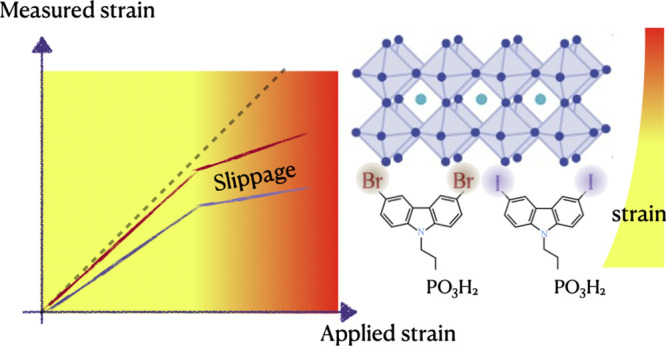

Hybrid organic–inorganic metal halide perovskites (MHP) are a class of semiconducting materials with potential applications in high-efficiency photovoltaics, light-emitting diodes, and photo- and X-ray detectors.^1–3^ Significant effort has been invested in developing MHP materials, resulting in a better understanding of how to tune the composition, modify interfaces, and optimize the hole and electron transport layers to improve optoelectronic properties for better device performance.^1,4–6^ For example, all-perovskite tandem solar cells have achieved >29% power conversion efficiency (PCE) by controlling the crystallographic orientation through the introduction of 2D templates.^7^ However, one major shortcoming preventing the commercial viability of MHP solar cells is degradation and phase transformation under normal operating conditions.^8–10^

Halide segregation is a major contributor to the instability and degradation of MHP alloys, and hence, it is important to deeply understand the mechanisms that contribute to this instability. One proposed mechanism for MHP photoinduced halide segregation is that lattice strain due to polaron formation upon absorption of light initiates halide migration and compositional segregation. This mechanism predicts that degradation is strongly dependent on the applied or intrinsic strain.^11,12^ It has been shown that applying compressive macrostrain can improve the chemical and phase stability of the MHPs.^13,14^ Increased compressive strain within MHPs also has been correlated to increased halide vacancy concentrations. Thus, it is important to better understand the impact of strain on MHP properties.^15–17^

Greater insight into this strain-dependent behavior requires characterizing the nature and extent of strain within MHPs, particularly the distinction between microstrain and macrostrain.^18^ Microstrain refers to local variations in a material’s lattice parameters at the microscopic or atomic scale, typically occurring within grains or across grain boundaries. It arises from nonuniform lattice distortions and manifests as broadening of diffraction peaks that depends on peak order. In contrast, macrostrain denotes the overall or average strain across a large volume of the material, representing uniform deformation that is observed as a shift in diffraction peak position.^19^ In mixed A-site and halide MHPs, microstrain can result from chemical and structural phase heterogeneity, which have a profound impact on device stability and phase transitions within the working temperature range.^20,21^ Due to MHP softness, macrostrain is induced by the coefficient of thermal expansion (CTE) mismatch between the MHP film and the substrate. The CTE of organic–inorganic hybrid MHPs is large (α ≈ 5 × 10^–5^ K^–1^) compared to traditional semiconductors, such as Si (2.6 × 10^–6^ K^–1^). Substrate materials commonly used in devices, such as glass and indium tin oxide (ITO), have CTEs of approximately α_*s*_ = 1 × 10^–6^ K^–1^ and 5 × 10^–6^ K^–1^ to 8 × 10^–6^ K^–1^, respectively.^22,23^ The large CTE mismatch between the MHP and substrate induces macrostrain during the device fabrication and in the operating environment (e.g., high and low temperature swings). Thus, strain engineering is a key approach for enhancing the PCE and stability of perovskite solar cells.^24^

Strain engineering has been widely used in tuning the performance of semiconductors. In Complementary Metal-Oxide-Semiconductor (CMOS) manufacturing, the transistor benefits from strain-boosting electron/hole mobility.^25^ In quantum dots (QD) solar cells, a strain compensation layer is used to increase the open circuit voltage (*V*_oc_).^26^ Researchers have also demonstrated that the optical properties of a monolayer can be controlled by strain engineering via CTE mismatch.^27^ Several methods have been used to manage the macrostrain and microstrain with a focus on improving the device stability and efficiency. These methods have included A-site alloying, reducing the annealing temperature, and interfacial engineering.^28–31^ However, strain engineering has not been widely applied at the device level in MHP solar cells compared to conventional semiconductor manufacturing. One fundamental assumption used in strain engineering is that the perovskite-transport layer interface has a rigid, mechanical coupled contact: the film strain is equal to an externally applied strain and is stable over time.^28^ However, this assumption has not been tested and may not hold due to the softness of the MHPs and complex redox reactions at the interface. A recent study shows that strain induced by the mismatch in CTE relaxes over time due to moisture uptake at grain boundaries.^32^

In this work, we use in situ device bending coupled with Grazing Incident Wide-Angle X-ray Scattering (GIWAXS) to understand how the applied macrostrain changes the biaxial MHP strain. We developed a quantitative method to determine the MHP-Hole Transport Layer (HTL) interfacial mechanical coupling, and we study the impact of inserting different interfacial layers between the perovskite and substrate and comparing their applied and measured strain response. Combining the bending and annealing, we present an in-depth characterization of macrostrain behavior in MHP thin films, which can serve as a foundation for further strain engineering in perovskite solar cells (PSCs).

We employ a device stack consisting of perovskite/interlayer/ITO/polyethylene terephthalate (PET) or glass. The perovskite composition used is Cs_0.1_FA_0.9_Pb­(I_0.83_Br_0.17_)_3_. This mixed-halide formulation was selected for its favorable device performance, as well as its chemical and photostability.^33^ This composition forms a cubic phase at room temperature, enabling accurate strain analysis by eliminating peak assignment uncertainty due to the tetragonal phase (see Supporting Information (SI)). As interlayers, we utilize self-assembled monolayers (SAMs) of [2-(9*H*-carbazol-9-yl)­ethyl] phosphonic acid (2PACz), and its chemical variants I-2PACz, and Br-2PACz, due to their widespread application in PSCs for enhancing PCE.^34–37^

We use a flexible substrate to induce macrostrain via bending. Because the substrate is much thicker than the other layers in the stack, the bending curvature determines the strain within the substrate. This strain is transferred to the MHP thin film, with the transfer efficiency governed by the in-plane mechanical coupling at the substrate–MHP interface. For clarity, this strain transfer is distinct from interfacial adhesion, which characterizes the resistance to delamination in the out-of-plane direction.^38^ Figure 1(a) shows the neutral axis bending model. The mechanical strain induced by bending can be described by^39^

1 $$\epsilon =\frac{h}{2r},h\ll r$$

where *h* is the thickness of the flexible substrate and *r* is the bending radius.^40^ This model considers a neutral axis in the middle of the flexible substrate with a fixed length. We use convex and concave bending to apply tensile and compressive strain, respectively. Figure 1(b) shows part of the in situ bending stage, where the MHP film is suspended between a fixed sample holder and a movable arm, controlled by a linear motor. We monitored the ITO film to keep the bending below the cracking level using Atomic Force Microscopy (AFM). The AFM images are included in the Supporting Information (Figures S12–S16). The strain levels follow the relation: natural deformation of the PET substrate (∼0.1%) < strain level investigated in this work (0.1–1.5%) < cracking/wrinkling threshold (∼2.5%). The radius of curvature is adjusted by varying the distance between the two holders. The flexible substrate adopts a catenary shape under free-standing conditions,

2 $$y=r\cdot \cosh\left(\frac{x}{r}\right)$$

where *x* and *y* are the coordinates of the side view plane and *r* is the bending radius at the top of the curve. The strain applied in the substrate is not uniform in this geometry, but the local strain is calculated using the local radius of curvature and eq 1.

**Figure 1:**
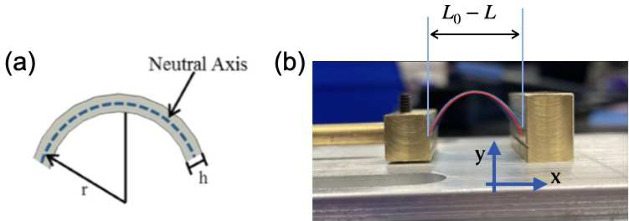
(a) Bending-strain model. *h* is the thickness of the substrate and *r* is the bending radius. Dashed line represents the neutral axis, which has a fixed length during the bending process. (b) Side view of the free-standing flexible substrate on the in situ bending stage. *L* represents the distance between the sample holders and *L*_0_ is the sample length. The substrate has a thickness of 177.8 μm and is hard to discern in the picture. The red curve is a fit of the bent substrate using the catenary function.

GIWAXS images are collected for different bending radii. The X-ray beam is perpendicular to the bending direction and aligned at the top/center of the films by controlling the sample horizontal position to make the reflected beam vertical. This ensures that *Q*_*z*_ is aligned with the out-of-plane direction and *Q*_*xy*_ is the in-plane direction, where *Q*_*z*_ is in the direction perpendicular to the thin film sample and *Q*_*xy*_ is parallel to the sample. Figure 2 illustrates the orientations of *Q*_*xy*_ and *Q*_*z*_ relative to the thin film.

**Figure 2:**
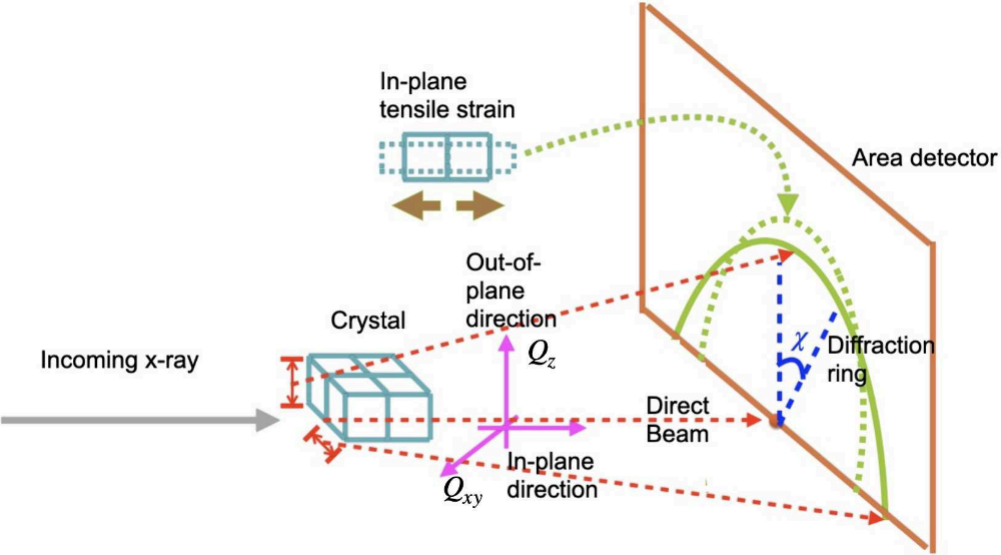
Diagram for strain measurement using GIWAXS. The synchrotron X-ray beam comes from the left side of the diagram. The thin film is placed parallel to the X-ray beam. The in-plane and out-of-plane directions are defined according to the top surface of the thin film. An area detector is placed downstream and perpendicular to the X-ray beam to collect the diffraction pattern. The solid and dashed rings on the detector indicate the diffraction pattern of the crystal with no strain and in-plane tensile strain, respectively.

We use GIWAXS to measure the diffraction rings from the MHP thin film. The diffraction rings are circular for a strain-free, polycrystalline material due to the isotropy of the lattice parameter. An imposed strain causes a uniaxial change in lattice parameter and a shift in diffraction ring position and shape. Isotropic strain induces an overall lattice parameter change and an isotropic diffraction peak shift. However, an in-plane strain causes an anisotropic lattice parameter change for in-plane and out-of-plane directions, and the diffraction ring distorts into an ellipse. Figure 2 shows a schematic diagram of the GIWAXS experiment for our strain measurements. The diffraction peak position in reciprocal space and the lattice parameter are related by Bragg’s law, $q=\frac{2\pi }{d}$ , where *d* is the lattice parameter and *q* is the diffraction peak position. The strain ϵ_χϕ_ in the (χ,ϕ) direction is,

3 $$\epsilon_{\chi \phi }=\frac{d_{0}-d_{\chi \phi }}{d_{0}}=\frac{1+\nu }{E}\sigma \cos^{2}\phi \sin^{2}\chi -\frac{\nu }{E}\sigma$$

where *E* is the modulus of elasticity, ν is the Poisson’s ratio, and σ is the uniaxial strain along the χ = 90°, ϕ = 0° direction, *d*_0_ and *d*_χϕ_ are the unstrained *d*-spacing and the strained *d*-spacing in the (χ,ϕ) direction. In GIWAXS, the angles ϕ and χ are related by the Ewald sphere geometry at fixed *Q* (Figure 2 and SI). The cos^2^ ϕ factor gives a constant shift in the ϵ vs sin^2^ χ (see SI). To determine the diffraction ring distortion with an accuracy of <0.05%, we performed experiments at a high *Q* resolution GIWAXS beamlines, 12-ID at NSLS-II, and 11-3 at SSRL.

The interfacial attachment between the MHP and the substrate forms at high temperatures during annealing. As the device is cooled to room temperature, a CTE mismatch develops between the perovskite and the substrate and induces strain in the film. However, it has been observed that this CTE mismatch-induced strain experiences a time-dependent decay after cooling to room temperature,^32^ but the strain does not reduce to zero or reverse sign. Thus, a residual strain is established in the MHP film and is typically tensile with most substrates due to the large CTE of perovskite compared to typical substrates.

To invert the sign of the residual strain from tensile to compressive, we designed a prestraining process. The MHP film is first spin-coated on a flexible PET substrate (Figure 3(1)), and then transferred to a curved sample holder. The film is fixed on a curved surface using a frame with an opening on its top to allow solvent evaporation (Figure 3(2)). The sample holder is then transferred to the hot plate for annealing (Figure 3(3)). At a high annealing temperature, a strain-free attachment develops between MHP and substrate in the curved geometry. When the film is released back to flat at room temperature, a compressive strain is applied to the MHP layer. Figure 3 shows the prestrain process and a photo of the curved sample holder.

**Figure 3:**
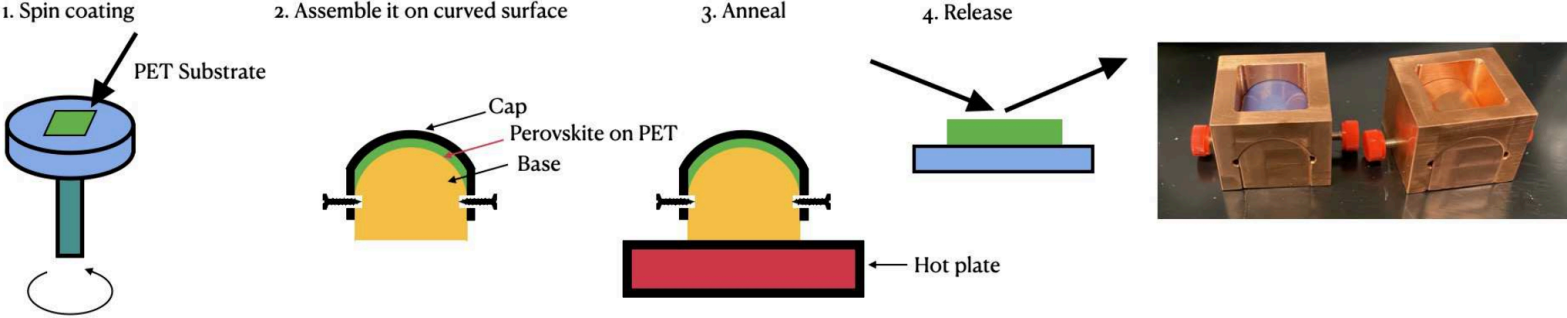
Prestrain process applied to the MHP film: (1) Spin-coating the MHP on a PET substrate; (2) Mounting the film onto a curved surface, the device is secured between a cap and a copper base, which provides good thermal conductivity; (3) Annealing the MHP thin film while held in the curved configuration; (4) Releasing the film and returning it to a flat state for testing. (right) Picture of the curved sample holder.

The interface between layers is crucial for the stability of PSCs. The complex nature of the perovskite-HTL interface and the MHP softness introduce uncertainty regarding the presence of interfacial slippage between the MHP layer and the underlying substrate. Slippage can decouple the mechanical response of the film from that of the substrate, thereby obviating the assumption of coherent strain transfer. As a result, the film strain may not reflect the strain imposed by the substrate, voiding assumptions made about strain induced by CTE mismatch. In our experiment, we use the applied strain from bending the flexible substrate compared to the measured strain to examine the mechanical attachment between the MHP layer and ITO or HTL. A perfect mechanical attachment results in the applied strain equaling the measured strain.

Figure 4(a) shows an in situ bending GIWAXS image collected at 12-ID-D, NSLS-II. The sharp diffraction rings are from the perovskite layer. The highly oriented diffraction peaks are from the PET substrate. Transmission WAXS is also captured due to the free-standing geometry. We use the (001) Bragg peak of the cubic Cs_0.1_FA_0.9_Pb­(I_0.83_Br_0.17_)_3_ for a sin^2^ χ analysis (eq 3) to determine the measured uniaxial strain. A sector mask of 5° is used to extract the diffraction peak position as a function of χ, as shown in Figure 4(b). The data in the sector mask is integrated over χ to give *I*(*Q*). An example is shown in Figure 4(c) along with a Gaussian function and a linear background that are used to fit these data to accurately determine the diffraction peak position. By performing this operation repeatedly along the diffraction ring, a *Q*(001) vs χ relationship is determined. The GIWAXS measurement captures both the left side (negative *Q*_*xy*_) and right (positive *Q*_*xy*_) sides of the diffraction ring (Figure 4(d)), which in principle, gives the same slope in sin^2^ χ analysis. In our experiment, we determined the systematic error of strain from the difference between the left and right sides of the images. The accuracy of strain measurement using the sin^2^ χ method is estimated to be ±0.07% based on the GIWAXS analysis. Both instrumental resolution and crystallite-level fluctuations are reflected in the scatter of the sin^2^ χ data shown in Figure 4(d). The instrumental resolution arises from a combination of detector *Q* resolution and detector geometry calibration. An error bar of ±0.05% (based on the left vs right side of the diffraction ring) is assigned to each data point in Figure 4(d) and the uncertainty in the fitted slope is ±0.07%.

**Figure 4:**
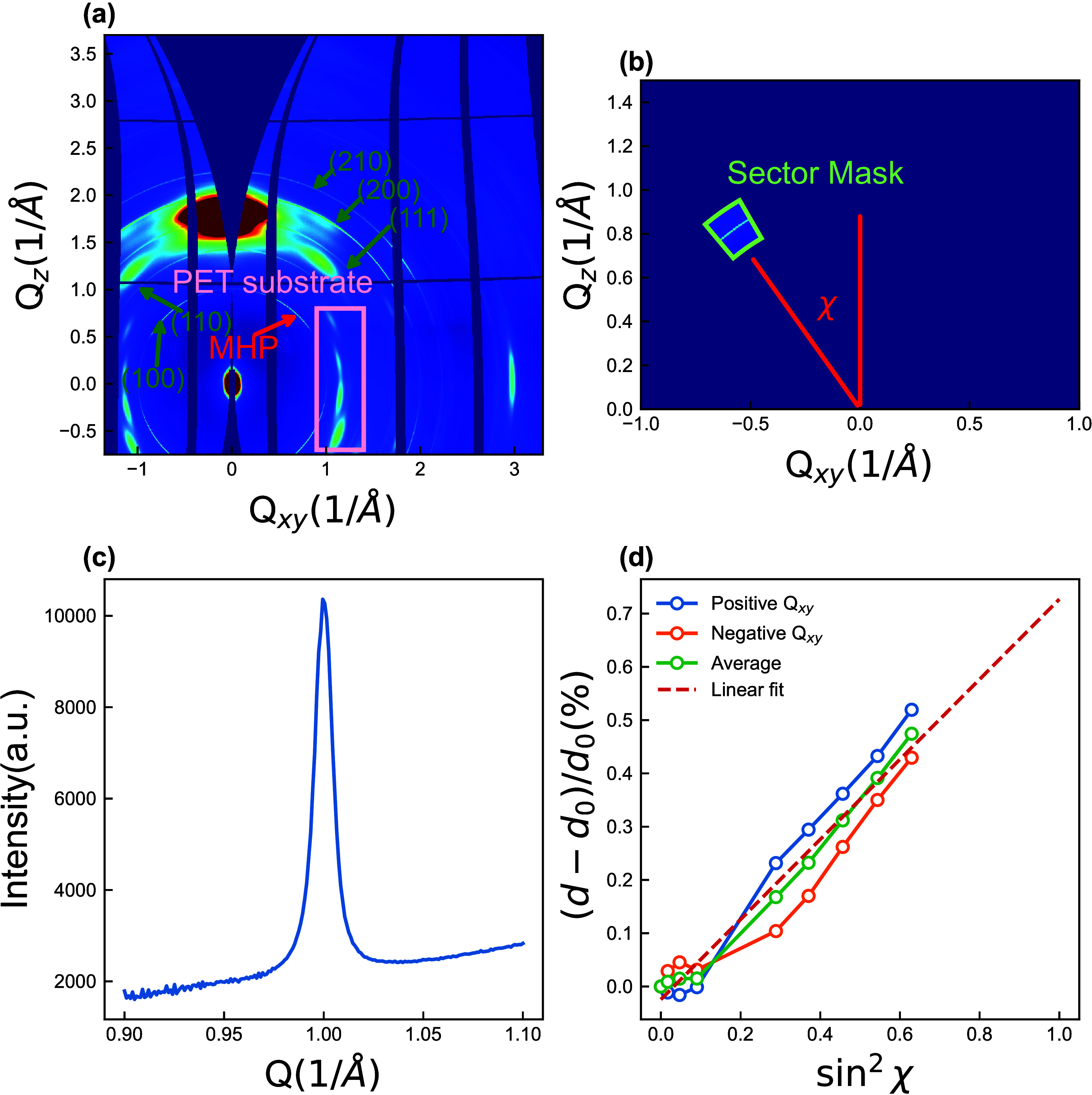
(a) GIWAXS pattern of the Cs^0.1^FA^0.9^Pb­(I^0.83^Br^0.17^)_3_-ITO-PET stack, collected at beamline 12-ID-D of NSLS-II using 12.7 keV X-rays and an incident angle of 4.5°. The image is stitched from two measurements taken at different detector positions to extend the *Q*-range. The sharp diffraction rings are from the MHPs and are indexed in green. The highly textured diffraction peaks are from the PET substrate. The use of free-standing samples enables the capture of low and negative *Q*_*z*_ signals by allowing X-ray transmission through the sample. Measurements were conducted under vacuum. (b) Sector mask used for azimuthal integration of the GIWAXS pattern, highlighting the region selected for extracting the intensity profile along the (001) diffraction peak. The sector mask has a χ range of 5°. (c) Sector mask integrated intensity as a function of scattering vector *Q*, showing the (001) diffraction peak along with the best fit. (d) sin^2^ χ analysis. The strain is calculated using the (001) diffraction peak position extracted from integrated GIWAXS at different χ. The blue and orange dots are the strain calculated from the positive *Q*_*xy*_ (right side of the diffraction ring) and negative *Q*_*xy*_ (left side of the diffraction ring), respectively. The green dots are the average strain of the two, and the dashed line is the linear fit of the average strain.

Figure 5 presents the relationship between applied strain and measured strain across different interfaces. We applied from 1.2% compressive strain to 0.8% tensile strain (−0.8% to 1.2%) to the MHP-interlayer-ITO-PET stack. On the tensile side, in the small strain regime (−0.4% to 0%), the measured strain of all four samples exhibits a linear response with slightly smaller than unity slope, indicating reasonably strong interfacial mechanical coupling. Beyond 0.4% tensile applied strain, a nonlinear response emerges, with the measured strain showing a gradual, sample-dependent reduction below the expected linear behavior (solid line) as the applied strain increases. This progressive decrease suggests some interface slippage between the perovskite and the substrate. On the compressive side, the strain response exhibits a similar trend, but the deviation from the applied strain is smaller in the low-strain regime and sample dependent. This slight asymmetry may stem from the grain boundaries responding differently to tensile and compressive strain, potentially due to variations in defect formation, grain boundary sliding, or moisture adsorption mechanisms under different strain states.

**Figure 5:**
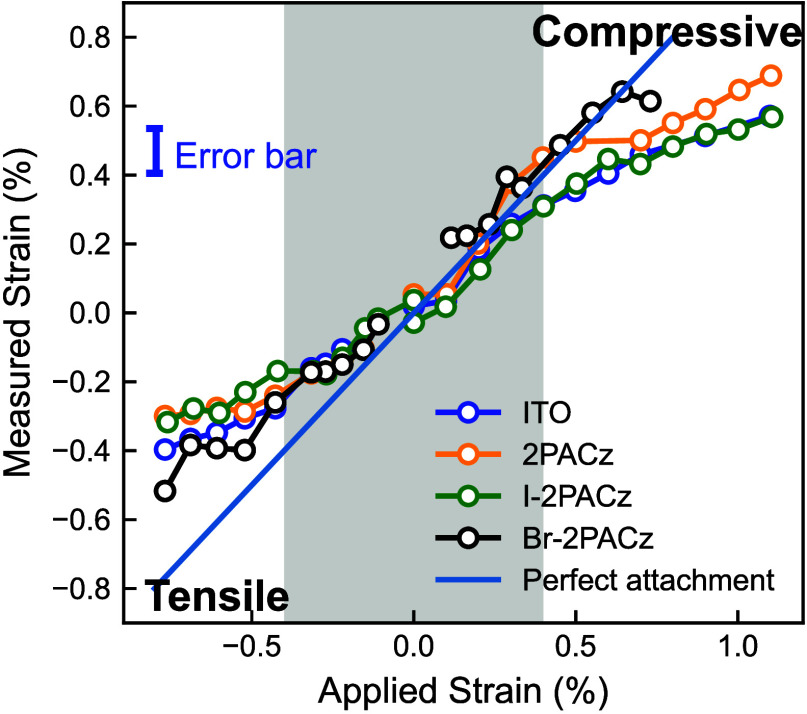
Measured strain versus applied strain for different interlayers in the perovskite/ITO/PET stack. Data are shown for devices with no interlayer (ITO, blue), 2PACz (orange), I-2PACz (green), and Br-2PACz (black). The solid blue line represents the ideal case of perfect mechanical attachment, where the measured strain equals the applied strain. Negative values correspond to tensile strain, while positive values correspond to compressive strain. All interfaces exhibit a linear response at low strain, followed by deviation due to interfacial slippage. Among the interlayers, Br-2PACz shows the strongest interfacial mechanical coupling, sustaining the linear regime over a wider strain range and exhibiting the smallest deviation at high tensile strain. The shaded area represents the linear-response region from −0.4% to 0.4% applied strain. The error bar for each data point is estimated to be ±0.07% and for visual clarity, a single representative error bar (in blue) is displayed on the left side of the figure.

We also varied the interlayers by using different SAMs to investigate whether interfacial slippage is influenced by their surface chemistry and bonding, which can affect mechanical coupling at the perovskite–substrate interface. Given their widespread use in PSCs to improve interfacial quality, charge transport, stability, and device performance, understanding the role of SAMs in slippage behavior is particularly important.^37,41,42^ In our experiment, 2PACz, I-2PACz, and Br-2PACz are inserted between ITO and the perovskite layer. The three SAM layers show a similar surface coverage on ITO.^43^ Figure 5 shows the strain response of different interlayers. Surprisingly, all three SAMs as well as the bare ITO, give qualitatively similar responses and slippage transition values as the applied strain increases. However, the quantitative linear-response range and strain reduction behavior are slightly different. Br-2PACz shows the strongest interfacial mechanical coupling among the three SAMs. The linear response regime extends beyond 0.5% applied strain, and the strain reduction at higher applied strains is significantly smaller, especially in the compressive regime.

Based on the slippage transitions observed for the different interlayers, we divide the strain response into three regions: a linear-response region (applied strain from −0.4% to 0.4%, shaded in Figure 5), a tensile-slippage region (applied strain > −0.4%), and a compressive-slippage region (applied strain < 0.4%). Linear fits were performed for each interlayer within each region to extract the corresponding slope of the applied versus measured strain. The slope reflects the degree of mechanical coupling at the interface, ranging from complete interfacial detachment (slope = 0) to perfect attachment (slope = 1). Fits for each sample and each region are provided in the Supporting Information. The strain induced by the CTE mismatch can be estimated to be approximately ± 0.25% for a 50 K operating temperature window and a CTE difference of ΔCTE ≈ 0.5 × 10^–4^ K^–1^. This strain range lies within the linear-response region under normal operating conditions, unless extreme temperatures or perovskites with unusually high CTEs are used. The slopes in the linear-response region for bare ITO, 2PACz, I-2PACz, and Br-2PACz are 0.68, 0.81, 0.69, and 0.82, respectively, indicating that even at small applied strains, approximately 25% of the strain is dissipated at the interface through slippage. In the tensile-slippage region, the slope values for bare ITO, 2PACz, and I-2PACz decrease to 0.33, 0.07, and 0.36, respectively; in the compressive-slippage region, the corresponding slopes are 0.37, 0.34, and 0.32. These values show that the interfacial slippage increases to roughly 65% of the applied strain once slippage occurs. Br-2PACz exhibits essentially no slope change beyond 0.4% on compression, indicating negligible slippage. The extracted slopes for all interlayers in the three strain-response regions are summarized in Table 1. Overall, these results show the following trend in interfacial attachment strength: I-2PACz ≤ bare ITO ≤ 2PACz ≤ Br-2PACz.

**Table 1:** Extracted Slopes of the Applied versus Measured Strain Curves for Devices with Different Interlayers^a^

Interlayer	Linear	Tensile	Comp.
No interlayer (bare ITO)	0.68	0.33	0.37
2PACz	0.81	0.07	0.34
I-2PACz	0.69	0.36	0.32
Br-2PACz	0.82	0.82	0.82

a ‘Linear’, ‘Tensile’, and ‘Comp.’ denote the slopes in the linear-response, tensile-slippage, and compressive-slippage regions, respectively.

The chemical bonding between MHPs and halogenated SAMs remains under active investigation. Density functional theory suggest that the halogen group in SAMs bonds to the uncoordinated Pb and halogen ions in MHPs with (001) orientation.^44^ For 2PACz and its halogenated derivatives, the SAMs-halogen ions interactions appear to be more dominant.^45^ X-ray reflectivity (XRR) measurements reveal a more complex behavior: the thickness of the SAMs layer is different in the 2PACz-MAPbI_3_ and 2PACz-MAPbBr_3_ interface.^46^ This indicates that 2PACz and its halogenated derivatives can form distinct interface morphologies with mixed-halide perovskites, including variations in interface defect density and the presence of unbound SAM molecules. These differences in interface properties, which are influenced by the halogen group in Br-2PACz, may modulate the strain slippage; for example, Br-2PACz is more hydrophobic,^47^ potentially reducing moisture uptake. However, slippage is also observed under vacuum, suggesting other mechanisms are involved. The halogen functional groups can also modify the defect distribution at the interface, as variations in halogen–halide interactions influence the formation of ionic defects and consequently affect ion migration within the MHP lattice.

The CTE-induced macrostrain that the MHP film experiences is a time-dependent decay after an initial annealing during film formation. The inverse process of this strain relaxation is observed when the film is reannealed: the tension in the perovskite film recovers to its prerelaxed value.^32^ A moisture uptake model was proposed to explain this strain behavior: stress relaxation occurs as water molecules enter the perovskite grain boundaries, leading to a reduction in internal stress and lattice expansion.^32^ Figure 6(b) shows a schematic illustration of the strain relaxation.

**Figure 6:**
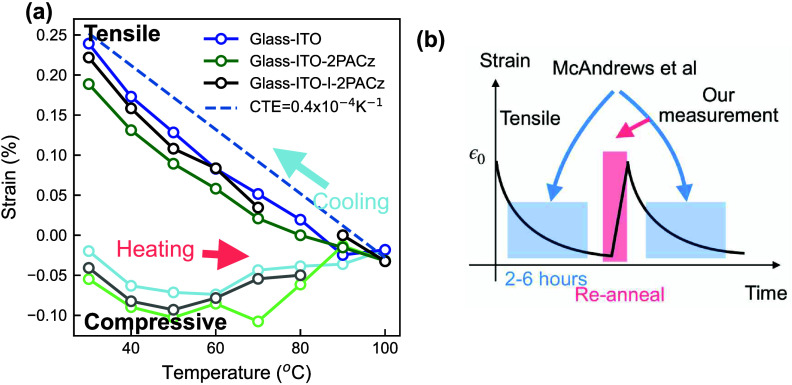
(a) Temperature-dependent strain behavior of MHP films on glass substrates with different interfacial layers: ITO only (blue), ITO/2PACz (green), and ITO/I-2PACz (black). For each case, the light-colored line indicates the heating phase (first half of the reannealing cycle), and the corresponding darker shade indicates the cooling phase (second half). (b) Schematic comparison between our measured strain recovery during reannealing and the previously reported time-dependent relaxation after cooling. The blue windows represent the 2–6 h time scale of strain relaxation. The red window highlights the reannealing step, during which strain is continuously recovered as the film is reannealed.

To quantitatively assess whether this reversible strain is induced by CTE mismatch and to determine if immediate slippage occurs as the strain develops, we measured the strain during the reannealing process using in situ GIWAXS. Immediate slippage refers to interfacial detachment that occurs as soon as strain begins to build, as shown in the applied strain versus measured strain experiment (Figure 5), while delayed slippage develops gradually over time. The MHP thin film on a glass substrate is placed in the GIWAXS chamber under an inert environment. We controlled the heating and cooling rates at 2.5 °C min^–1^ and GIWAXS measurements were taken every 10 °C. Figure 6(a) shows a temperature cycle during the reannealing process. The perovskite film was prepared 48 h prior to the measurement and stored in nitrogen gas; any strain had relaxed and the film is in steady state. Initially, the measured strain is slightly compressive and close to zero. During the heating half of the temperature cycle, the film is heated from 30 to 100 °C, but no significant changes in strain are observed with a slight compressive strain of about 0.05 to 0.1% independent of temperature between 40 to 80 °C. This behavior shows interface slippage, indicating that the perovskite layer does not remain rigidly attached to the substrate, perhaps due to an interfacial rearrangement process.

McAndrews et al. show that this CTE mismatch-induced strain is reversible over multiple cycles of reannealing (Figure 6(b)).^32^ However, the expected magnitude of the strain is not observed, due to the lack of time-resolved measurements during the reannealing process. In our experiment, the strain during cooling shows a linear increase in tensile strain from 100 to 30 °C, and the slope shows a good agreement with that expected from the CTE mismatch, as shown in Figure 6. Combined with the multicycle strain measurement,^32^ our in situ temperature experiment indicates that the interface readheres at high temperatures during the reannealing, and there is no immediate slippage. The strain caused by the CTE mismatch changes smoothly with temperature during reannealing, suggesting that no major reactions or phase transformations occur at the interface at higher temperatures. Unlike the case of applied strain versus measured strain shown in Figure 5, the strain behavior during reannealing is unaffected by different SAM interlayers between the perovskite layer and the glass substrate. This suggests that the weakening of the mechanical attachment between SAMs and perovskites accumulates during the strain relaxation process (blue window in Figure 6(b)), rather than resulting from inherently weak static interfacial chemical bonding. As strain relaxes, the SAM–MHP interaction weakens, indicating a gradual decoupling at the interface. The functional groups of the SAMs, such as I and Br, exhibit varying resistance to this weakening, reflecting differences in their interfacial attachment at room temperature.

Earlier, we demonstrated that macrostrain, introduced through bending or CTE mismatch during reannealing, can lead to slippage and interface rearrangement in perovskite films. To combine both bending and CTE mismatch impacts, we designed a prestrain process that applies compressive strain during annealing, aiming to counteract and reverse the tensile strain typically induced by CTE mismatch. The perovskite thin film was placed on a convex sample holder, introducing approximately 1% compressive strain—substantially greater than the 0.2% residual strain from CTE mismatch. Unlike bending at room temperature, annealing on a curved geometry induces plastic deformation of the PET substrate. To enable postannealing measurements, we used a customized sample holder that constrained the sample to remain flat. The film is strain-free in the deformed, curved geometry after annealing, and the external strain is introduced when it is subsequently constrained to lie flat.

We measured the residual strain 48 h after annealing. While the control sample (without prestrain) exhibited a small tensile strain, the prestrained sample retained a net compressive strain. This confirms that the prestrain process induces a compressive stress in the film, even after partial relaxation. To investigate how this prestrain relaxes, we reannealed another film on the curved holder (equivalent to prestrain 1% compression) in a glovebox and rapidly transferred the sample to the GIWAXS beamline. The first measurement, taken 30 min after annealing, showed a compressive strain of 0.5%. Over the next 4 h in vacuum, this strain gradually decreased to 0.3%. Table 2 shows the residual strain of prestrained MHP thin films. This behavior is similar to the relaxation observed for tensile strain from CTE mismatch, showing that compressive bending strain also undergoes relaxation.

**Table 2:** Residual Strain of Prestrained MHP Thin Films

Process	Tensile/Compressive	Strain
Flat annealing	Tensile	<0.2%
Prestrain 1%, 30 min after annealing	Compressive	0.5%
Prestrain 1%, 4 h after annealing	Compressive	0.3%
Prestrain 1%, relaxed to the steady state	Compressive	<0.2%

In this work, we systematically investigated the strain behavior in perovskite thin films under mechanical bending using in situ GIWAXS. Our findings reveal a nonlinear strain response at the perovskite-substrate interface, attributed to slippage beyond a critical applied strain of about 0.4%. By introducing different interlayers, we demonstrated that interfacial mechanical coupling plays a crucial role in macrostrain propagation with Br-2PACz exhibiting the strongest attachment. Furthermore, we explored the strain relaxation and rearrangement during the reannealing process, confirming that the macrostrain undergoes time-dependent relaxation but can be rearranged upon reheating. To leverage strain engineering for improved device stability, we implemented a prestrain process, where the perovskite was annealed on a curved substrate to introduce a controlled residual compressive strain. This approach successfully altered the residual strain state, demonstrating its potential as a strategy to modulate mechanical stability in perovskite solar cells.

Overall, our study provides critical insights into the strain relaxation and dynamics at perovskite interfaces, demonstrating some extent of interface slippage that should be accounted for when modeling CTE effects in PSCs. Our work offers a foundation for future strain-engineering strategies aimed at enhancing both stability and performance in flexible perovskite-based devices. Future work could examine the generality of our findings for different MHP compositions as well as establish the molecular mechanisms associated with interfacial mechanical coupling and slippage.

## Supplementary Material



## Notes

Detailed experimental procedures, theoretical derivations, and additional data supporting the main text. It includes the fabrication of SAM-modified ITO substrates and perovskite thin films, along with molecular structures of the interlayers. A catenary model is introduced to determine bending radius from substrate geometry. The relationship between applied mechanical strain and the strain measured by GIWAXS is derived by transforming stress tensors into the scattering vector coordinate system, with corrections accounting for measurement geometry. Additional analysis justifies the use of a cubic perovskite phase to avoid artifacts from tetragonal distortion. Experimental details of GIWAXS calibration and repeatability confirm measurement reliability. Finally, AFM and SEM characterization demonstrate that ITO cracking occurs only under extreme bending, ruling it out as the origin of strain reduction (PDF)
