# Supplementary material for: Strain in Metal Halide Perovskite Thin Films - Interfacial Mechanical Coupling
Source: ACS Energy Lett. 2026 May 14;11(6):4428–36. doi: 10.1021/acsenergylett.6c00526 (PMC13270641; doi:10.1021/acsenergylett.6c00526)
Supplement: Supplementary file 1 [file nz6c00526_si_001.pdf]

Supplemental Information for:

Strain in Metal Halide Perovskite Thin Films - Interfacial Mechanical Coupling

Zihan Zhang,[1] Collin A. Sindt,[2] Gabriel R. McAndrew,[3] Samantha C. Kaczaral,[2] Wenhan Ou,[3] Nicholas J. Weadock,[3] Michael D. McGehee,[2,3,4] and Michael F. Toney[2,3,4]

[1]*Department of Physics, University of Colorado Boulder, Boulder, CO 80309, USA*

[2]*Department of Chemical and Biological Engineering, University of Colorado Boulder, Boulder, CO 80309, USA*

[3]*Materials Science and Engineering Program, University of Colorado Boulder, Boulder, CO 80309, USA*

[4]*Renewable and Sustainable Energy Institute (RASEI), University of Colorado Boulder, Boulder, CO 80309, USA*

(Dated: May 3, 2026)

I. SAMPLE PREPARATION

We deposit self-assembled monolayer (SAMs) of [2-(9H-carbazol-9-yl)ethyl] phosphonic acid (2PACz), and its chemical variants I-2PACz, and Br-2PACz on ITO-Glass/PET substrates. Fig S1 shows the molecular structure for 2PACz, I-2PACz, and Br-2PACz. Substrates were first cleaned using sequential ultrasonic cleaning in 1 % Solujet phosphate-free detergent in deionized water, deionized water, and isopropyl alcohol for 10 minutes each. Between each sonication, the substrates were thoroughly dried using a dry nitrogen gun. Samples were UV-Ozone treated for 15 minutes to functionalize the surface, followed by transfer to an ambient spin coater and immediate deposition of each respective solution of phosphonic acid (1 mM in absolute ethanol for each molecule). Each 2PACz solution was heated to 35 °C and sonicated for 5 minutes immediately prior to deposition to minimize the influence of aggregation in solution. The casting solution was allowed to rest on the surface of the UV-Ozone treated substrates for 30 seconds before being spun at 3000 rpm for 30 seconds. Samples were then annealed in air at 100 °C for 10 minutes. Following annealing, samples were allowed to cool to room temperature, before being returned to the spin coater for a dynamic wash. Samples were spun at 6000 rpm for 1 minute, during which 3 x 150 microliter volumes of ethanol were pipetted onto the sample to remove overlayer phosphonic acid material, which may have accumulated. The sample was then again annealed at 100 °C for 10 minutes before being transferred to a glove box for perovskite deposition.

The $Cs_{0.1}FA_{0.9}Pb(Br_{17}I_{0.83})_3$ thin films were fabricated via spin-coating. Stoichiometric ratios of CsI, FAI, $PbBr_2$, and PbI_2 were measured to give a 1 M solution (1 mmol perovskite salts in 1 ml solvent. The salts were dissolved in a mixed of 4:1 (volumetric basis) DMF:DMSO and were stirred at room temperature for at least 24 hours prior to fabrication. On each substrate, 70 µL of $Cs_{0.1}FA_{0.9}Pb(Br_{17}I_{0.83})_3$ solution was deposited onto the center of the substrate. The substrate was then spun at 1,000 rpm for 10 s, 6,000 rpm for 30 s, and decelerated. On each substrate, at the optimized time, between 5 and 10 seconds remaining in the second spin step, 250 μL of chlorobenzene antisolvent was dropped onto the sample. The substrates were annealed 60 °C for 1 min and then at 105 °C for 30 minutes.

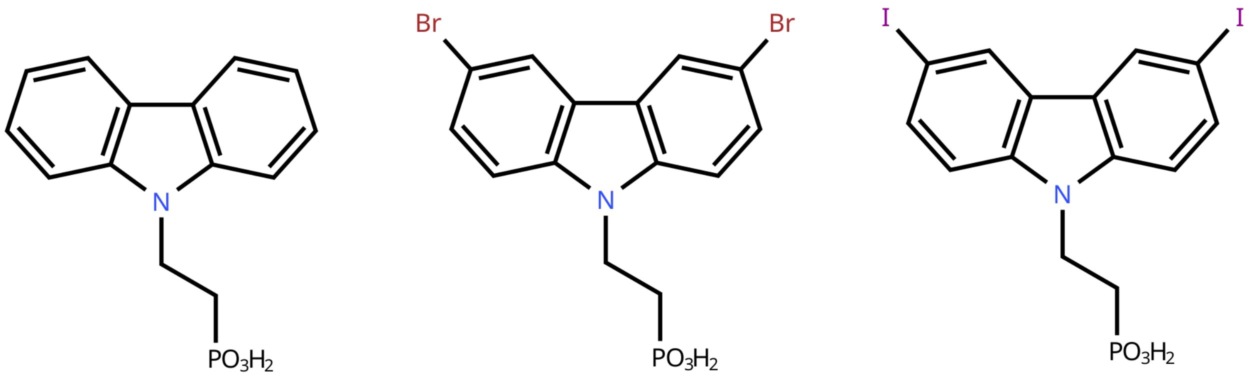

FIG. S1: The molecular structure of 2PACz, Br-2PACz, and I-2PACz.

II. CATENARY CURVE

The compressed free-standing flexible substrates form a catenary curve in space (Fig. S2),

$$y = r \cdot \cosh \frac{x}{r} = r \cdot \frac{e^{\frac{x}{r}} + e^{-\frac{x}{r}}}{2}, \tag{1}$$

where y is the vertical coordinate and x is the horizontal, r is a parameter with the units of length. The bending radius on the top of the curve is

$$R = \frac{(1 + (\frac{dy}{dx})^2)^{3/2}}{d^2y/dx^2} = r, \tag{2}$$

where $\frac{dy}{dx}\Big|_{x=0} = 0$, $\frac{d^2y}{dx^2}\Big|_{x=0} = \frac{1}{r}$.

$$\frac{dy}{dx} = \sinh \frac{x}{r} = \frac{1}{2}(e^{\frac{x}{r}} - e^{-\frac{x}{r}}) \tag{3}$$

$$\sqrt{dy^2 + dx^2} = \frac{1}{2}(e^{x/r} + e^{-x/r})dx \tag{4}$$

The length of the substrate is L_0:

$$\int_0^{L/2} \sqrt{dy^2 + dx^2} = \frac{1}{2}\int_0^{L/2} e^{x/r} + e^{-x/r}dx = \frac{r}{2}(e^{\frac{L}{2r}} - e^{-\frac{L}{2r}}) = \frac{L_0}{2} \tag{5}$$

$$r(e^{\frac{L}{2r}} - e^{-\frac{L}{2r}}) = L_0 \tag{6}$$

Thus, the bending radius can be obtained from the distance between sample holders, L, and the length of the substrate, L_0.

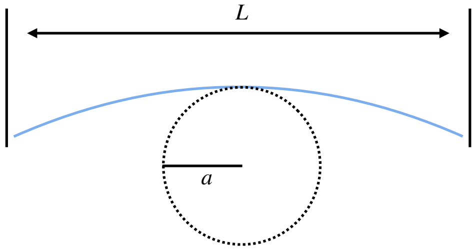

FIG. S2: Catenary curve.

III. STRESS TENSOR IN SPHERICAL COORDINATES

X-ray diffraction measures the lattice parameter in the direction of the scattering vector, **Q**. However, bending and CTE mismatch apply the macrostrain in the in-plane direction to the MHP thin film. The following section derives the relationship between the uniaxial/biaxial strain and the strain in the **Q** ((θ,ϕ) direction in spherical coordinates, Fig S3) direction.

The rotation matrices in Cartesian coordinates are

$$R_x(\theta) = \begin{pmatrix} 1 & 0 & 0 \\ 0 & \cos\theta & -\sin\theta \\ 0 & \sin\theta & \cos\theta \end{pmatrix}, \tag{7}$$

$$R_y(\theta) = \begin{pmatrix} \cos\theta & 0 & \sin\theta \\ 0 & 1 & 0 \\ -\sin\theta & 0 & \cos\theta \end{pmatrix}, \tag{8}$$

$$R_z(\theta) = \begin{pmatrix} \cos\theta & -\sin\theta & 0 \\ \sin\theta & \cos\theta & 0 \\ 0 & 0 & 0 \end{pmatrix}, \tag{9}$$

where $R_x(\theta)$, $R_y(\theta)$, $R_z(\theta)$, are the matrices representing a clockwise rotation by angle θ about the x, y, and z axes, respectively.

The uniaxial strain tensor takes the form:

$$\overleftrightarrow{\sigma} = \sigma^{\alpha\beta} = \begin{pmatrix} \sigma & 0 & 0 \\ 0 & 0 & 0 \\ 0 & 0 & 0 \end{pmatrix}. \tag{10}$$

Fig S3 shows the scattering vector. The stress tensor in the new Cartesian coordinate (x', y', z') is,

$$\sigma' = R_y^{-1}(\theta) R_z^{-1}(\phi) \overleftrightarrow{\sigma} R_z(\phi) R_y(\theta). \tag{11}$$

$$R_z^{-1}(\phi) \overleftrightarrow{\sigma} R_z(\phi) = \begin{pmatrix} \cos\phi & \sin\phi & 0 \\ -\sin\phi & \cos\phi & 0 \\ 0 & 0 & 1 \end{pmatrix} \begin{pmatrix} \sigma & 0 & 0 \\ 0 & 0 & 0 \\ 0 & 0 & 0 \end{pmatrix} \begin{pmatrix} \cos\phi & -\sin\phi & 0 \\ \sin\phi & \cos\phi & 0 \\ 0 & 0 & 1 \end{pmatrix} \tag{12}$$

$$= \begin{pmatrix} \sigma\cos^2\phi & -\sigma\sin\phi\cos\phi & 0 \\ -\sigma\sin\phi\cos\phi & \sigma\sin^2\phi & 0 \\ 0 & 0 & 0 \end{pmatrix} \tag{13}$$

$$R_y^{-1}(\theta) R_z^{-1}(\phi) \overleftrightarrow{\sigma} R_z(\phi) R_y(\theta) \tag{14}$$

$$= \begin{pmatrix} \cos\theta & 0 & -\sin\theta \\ 0 & 1 & 0 \\ \sin\theta & 0 & \cos\theta \end{pmatrix} \begin{pmatrix} \sigma\cos^2\phi & -\sigma\sin\phi\cos\phi & 0 \\ -\sigma\sin\phi\cos\phi & \sigma\sin^2\phi & 0 \\ 0 & 0 & 0 \end{pmatrix} \begin{pmatrix} \cos\theta & 0 & \sin\theta \\ 0 & 1 & 0 \\ -\sin\theta & 0 & \cos\theta \end{pmatrix} \tag{15}$$

$$= \begin{pmatrix} \sigma\cos^2\theta\cos^2\phi & -\sigma\cos\theta\sin\phi\cos\phi & \sigma\cos^2\phi\sin\theta\cos\theta \\ -\sigma\cos\theta\sin\phi\cos\phi & \sigma\sin^2\phi & -\sigma\sin\phi\cos\phi\sin\theta \\ \sigma\cos^2\phi\sin\theta\cos\theta & -\sigma\sin\phi\cos\phi\sin\theta & \sigma\sin^2\theta\cos^2\phi \end{pmatrix} \tag{16}$$

The strain in the (θ,ϕ) direction is

$$\begin{aligned} \epsilon &= \frac{1}{E}\sigma'_{33} - \frac{\nu}{E}(\sigma'_{22} + \sigma'_{11}) \\ &= \frac{1}{E}\sigma\sin^2\theta\cos^2\phi - \frac{\nu}{E}(\sigma\sin^2\phi + \sigma\cos^2\theta\cos^2\phi) \\ &= \frac{1+\nu}{E}\sigma\sin^2\theta\cos^2\phi - \frac{\nu}{E}(\sigma\sin^2\theta\cos^2\phi + \sigma\sin^2\phi + \sigma\cos^2\theta\cos^2\phi) \\ &= \boxed{\frac{1+\nu}{E}\sigma\sin^2\theta\cos^2\phi - \frac{\nu}{E}\sigma}. \end{aligned} \tag{17}$$

The CTE mismatch-induced biaxial strain is, by definition, invariant under rotation in the x-y plane:

$$\overleftrightarrow{\sigma_B} = \sigma_B^{\alpha\beta} = \begin{pmatrix} \sigma & 0 & 0 \\ 0 & \sigma & 0 \\ 0 & 0 & 0 \end{pmatrix}. \tag{18}$$

In the Cartesian coordinate (x', y', z'), the biaxial stress tensor becomes

$$\sigma' = R_y^{-1}(\theta) R_z^{-1}(\phi) \overleftrightarrow{\sigma_B} R_z(\phi) R_y(\theta) = R_y^{-1}(\theta) \overleftrightarrow{\sigma_B} R_y(\theta) \tag{19}$$

$$= \begin{pmatrix} \cos\theta & 0 & -\sin\theta \\ 0 & 1 & 0 \\ \sin\theta & 0 & \cos\theta \end{pmatrix} \begin{pmatrix} \sigma & 0 & 0 \\ 0 & \sigma & 0 \\ 0 & 0 & 0 \end{pmatrix} \begin{pmatrix} \cos\theta & 0 & \sin\theta \\ 0 & 1 & 0 \\ -\sin\theta & 0 & \cos\theta \end{pmatrix} \tag{20}$$

$$= \begin{pmatrix} \sigma\cos^2\theta & 0 & \sigma\sin\theta\cos\theta \\ 0 & \sigma & 0 \\ \sigma\sin\theta\cos\theta & 0 & \sigma\sin^2\theta \end{pmatrix} \tag{21}$$

The strain in the (θ,ϕ) direction is

$$\begin{aligned} \epsilon_B &= \frac{1}{E}\sigma'_{33} - \frac{\nu}{E}(\sigma'_{22} + \sigma'_{11}) \\ &= \frac{1}{E}\sigma\sin^2\theta - \frac{\nu}{E}\sigma(1 + \cos^2\theta) \\ &= \frac{1+\nu}{E}\sigma\sin^2\theta - \frac{\nu}{E}\sigma(\sin^2\theta + 1 + \cos^2\theta) \\ &= \boxed{\frac{1+\nu}{E}\sigma\sin^2\theta - \frac{2\nu}{E}\sigma}. \end{aligned} \tag{22}$$

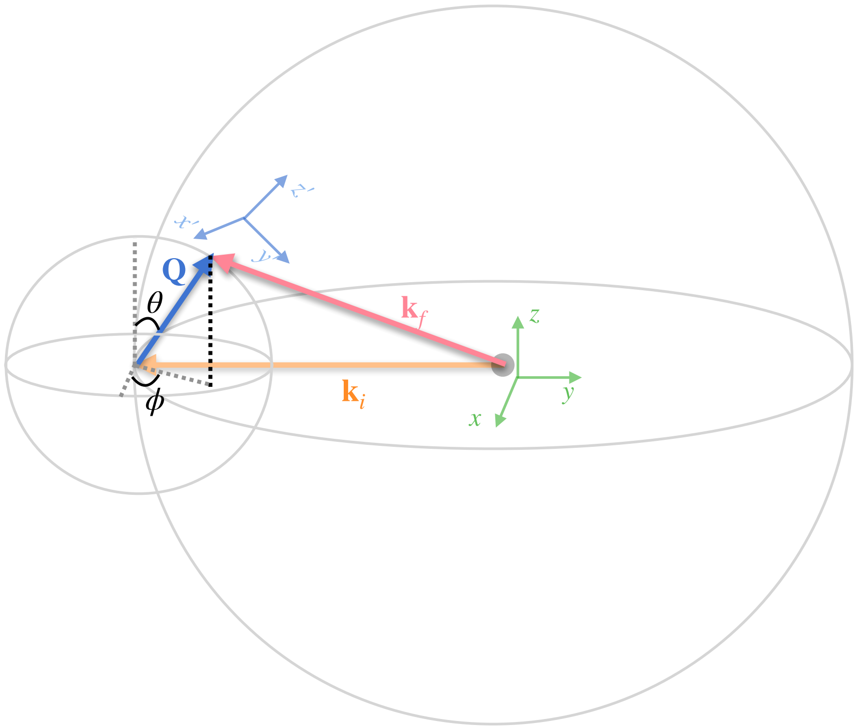

FIG. S3: The x-ray scattering sphere in GIWAXS.

IV. $\cos^2\phi$ CORRECTION IN GIWAXS GEOMETRY

In GIWAXS measurement, the spin-coated sample is in-plane isotropic. However, the bending applies an in-plane anisotropic strain. Fig S3 shows the geometry of GIWAXS. In our setup, the strain induced by bending is primarily in the x direction. The scattering vector $\mathbf{Q}$ has a non-zero ϕ in the spherical coordinate. This means the strain we measured in the Q_{xy} direction no longer reflects the applied uniaxial strain, unlike the case of biaxial strain. Thus, it is necessary to resolve the $\mathbf{Q}$ along the Q_x and Q_y directions. The following section shows that the correction from non-zero ϕ gives a constant shift in the $\frac{d-d_0}{d_0}$ vs $\sin^2\chi$ relation and does not affect the slope.

In Cartesian coordinates, the Q vector of incident X-ray, $\mathbf{k}_i$, scattered X-ray, $\mathbf{k}_f$, and the

scattering vector $\mathbf{Q}$ can be expressed as,

$$\mathbf{k}_i = \begin{pmatrix} -q \\ 0 \\ 0 \end{pmatrix}, \quad \mathbf{k}_f = \begin{pmatrix} q_f^x \\ q_f^y \\ q_f^z \end{pmatrix}, \quad \mathbf{Q} = \begin{pmatrix} q + q_f^x \\ q_f^y \\ q_f^z \end{pmatrix}. \tag{23}$$

The elastic scattering gives:

$$(q_f^x)^2 + (q_f^y)^2 + (q_f^z)^2 = q^2. \tag{24}$$

Define angle χ and ϕ as the polar angle and azimuthal angle of vector $\mathbf{Q}$.

$$\sin\phi = \frac{q + q_f^x}{|\mathbf{Q}|\sin\chi} \tag{25}$$

$$\begin{aligned}
(q_f^x + q)^2 + (q_f^y)^2 + (q_f^z)^2 &= |\mathbf{Q}|^2 \\
(q_f^x)^2 + (q_f^y)^2 + (q_f^z)^2 + q^2 + 2qq_f^x &= |\mathbf{Q}|^2 \\
2q^2 + 2qq_f^x &= |\mathbf{Q}|^2 \\
2q(q + q_f^x) &= |\mathbf{Q}|^2 \\
q_f^x + q &= \frac{|\mathbf{Q}|^2}{2q}
\end{aligned}$$

$$\sin\phi = \frac{q + q_f^x}{|\mathbf{Q}|\sin\chi} = \frac{|\mathbf{Q}|}{2q\sin\chi} \tag{26}$$

$$\cos^2\phi = 1 - \frac{|\mathbf{Q}|^2}{4q^2\sin^2\chi} = 1 - \frac{\lambda^2|\mathbf{Q}|^2}{16\pi^2\sin^2\chi} \tag{27}$$

The $\sin^2\chi\cos^2\phi$ becomes $\sin^2\chi - \frac{|\mathbf{Q}|^2}{4q^2}$, which gives a constant shift on $\frac{d-d_0}{d_0}$ vs $\sin^2\chi$ relation. The slope of $\frac{d-d_0}{d_0}$ vs $\sin^2\chi$ remains the same.

V. ORIENTED TETRAGONAL PHASE

We chose the MHP composition of $Cs_{0.1}FA_{0.9}Pb(I_{0.83}Br_{0.17})_3$ in our experiment. This composition forms a cubic phase at room temperature and above. The cubic phase ensures an accurate strain analysis by eliminating the impact of the oriented tetragonal phase that can confuse the (002) and (110) (tetragonal unit cell) due to their close Q positions. If the tetragonal phase crystal exhibits a preferred orientation, the difference between the tetragonal lattice parameters a and c will manifest in the in-plane and out-of-plane directions in GIWAXS, introducing bias into the strain analysis. Even if the film is completely isotropic, the (001) will be broadened, making the measurement less accurate. Fig S4 shows a diagram of the GIWAXS measurement for the crystal in the tetragonal phase with preferred orientation.

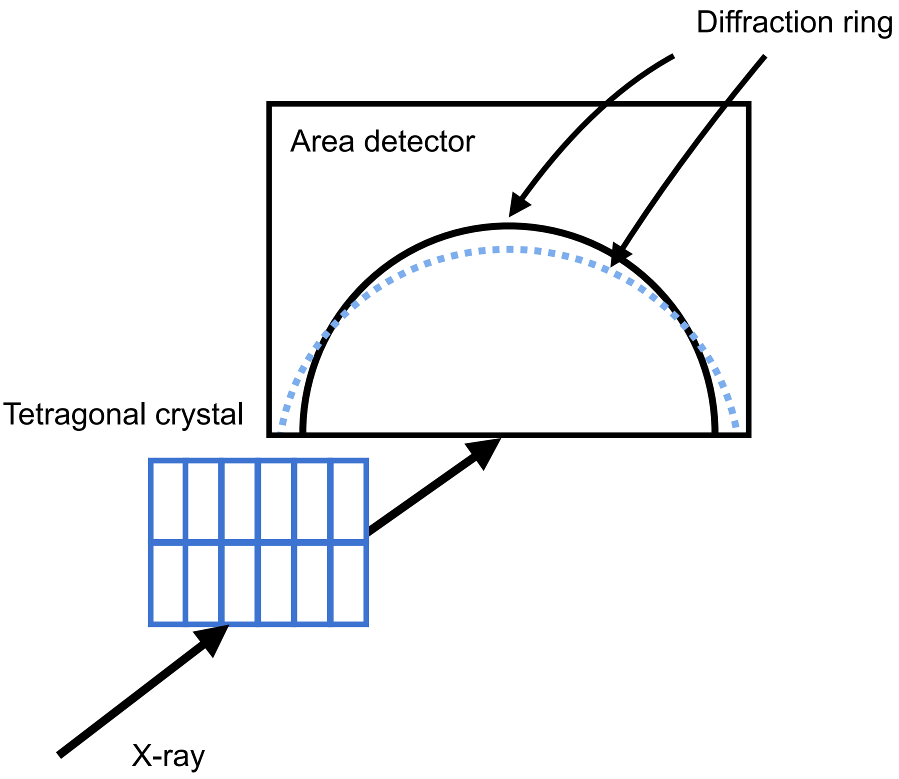

FIG. S4: Schematic of the GIWAXS measurement setup. It assumes that the tetragonal phase crystal has a preferred orientation, with the lattice parameter c primarily aligned along the out-of-plane direction. As a result, the diffraction peak shifts to higher Q values in the in-plane direction and to lower Q values in the out-of-plane direction. The solid and dashed lines represent diffraction rings for the cubic and oriented tetragonal phase, respectively.

VI. LINEAR FITS FOR APPLIED VERSUS MEASURED STRAIN CURVES

In this work, we characterized the relationship between the applied strain and the measured strain by bending the devices to different radii of curvature. The device stack consists of PET/ITO/interlayer/perovskite. The resulting strain–strain curves can be divided into three regions. (1) Linear-response region (applied strain between –0.4% and 0.4%): in this central region, the measured strain exhibits the highest and nearly constant sensitivity, with slopes typically ranging from 0.65 to 0.85. The boundary between linear-response and slippage at ±0.4% is approximate; the exact limits vary slightly between samples and are defined

by the point at which the slope begins to deviate from linearity. (2) Slippage regions at both the tensile and compressive sides (applied strain $< -0.4\%$ or $> 0.4\%$): interfacial slippage becomes more pronounced, leading to a reduced and nonlinear strain response.

We present the linear fits for the applied versus measured strain curves in each of these regions. Figure S5-S8 compares the results for devices with different interlayers—2PACz, I-2PACz, Br-2PACz, and a control without an interlayer.

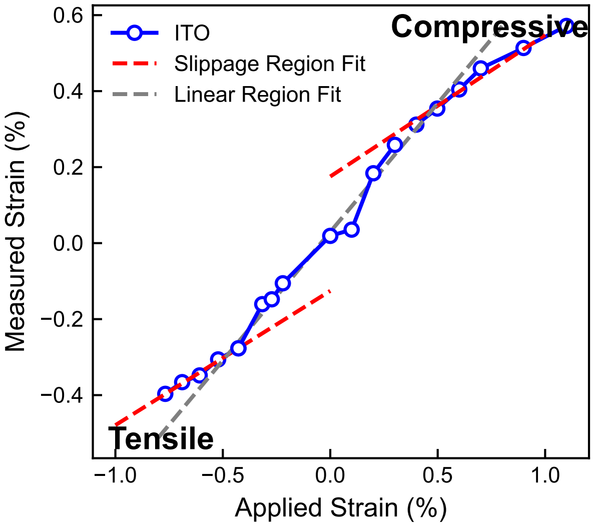

FIG. S5: Applied versus measured strain for PET/ITO/perovskite. Blue dots represent the experimental data. The dashed gray and red curves show the linear fits for the linear-response region and the tensile/compressive slippage regions, respectively. The extracted slopes are 0.68 in the linear-response region, 0.33 in the tensile slippage region, and 0.37 in the compressive slippage region.

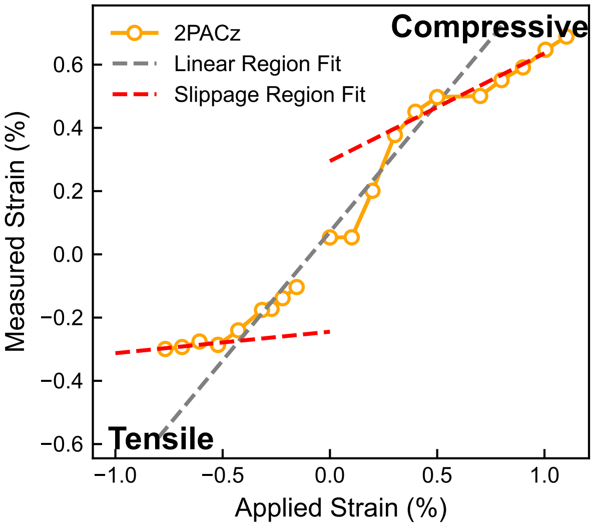

FIG. S6: Applied versus measured strain for PET/ITO/2PACz/perovskite. Orange dots represent the experimental data. The dashed gray and red curves show the linear fits for the linear-response region and the tensile/compressive slippage regions, respectively. The extracted slopes are 0.81 in the linear-response region, 0.07 in the tensile slippage region, and 0.34 in the compressive slippage region.

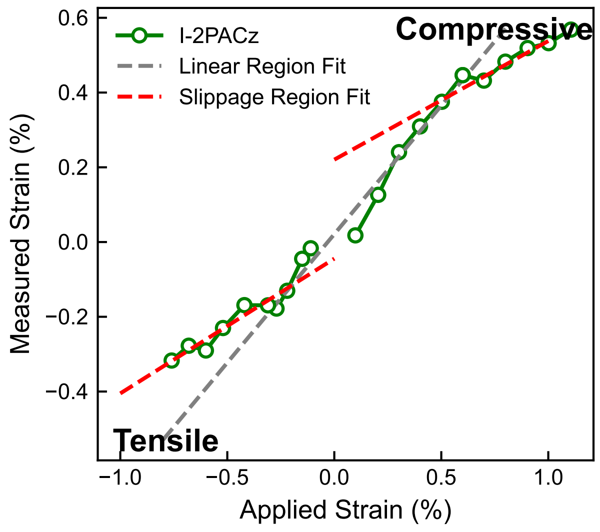

FIG. S7: Applied versus measured strain for PET/ITO/I-2PACz/perovskite. Green dots represent the experimental data. The dashed gray and red curves show the linear fits for the linear-response region and the tensile/compressive slippage regions, respectively. The extracted slopes are 0.69 in the linear-response region, 0.36 in the tensile slippage region, and 0.32 in the compressive slippage region.

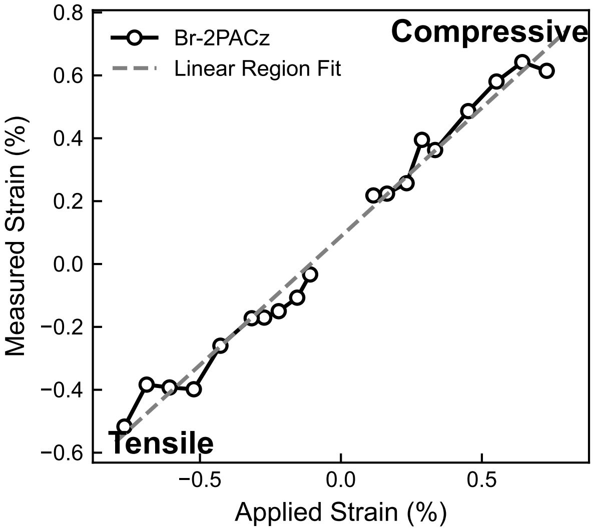

FIG. S8: Applied versus measured strain for PET/ITO/Br-2PACz/perovskite. Black dots represent the experimental data. The dashed gray curve shows the linear fit across the entire dataset. No noticeable slippage is observed at high applied strain for the Br-2PACz interlayer. The slope is 0.82.

VII. GIWAXS BEAMLINE CALIBRATION

The in-situ bending GIWAXS experiments are performed at 12-ID, NSLS-II, Brookhaven National Lab. The detector position is calibrated using AgBh before the measurement. We also use the sharp diffraction rings from perovskite to locate the beam center for each GIWAXS measurement to prevent the drift of the beam or instrument during the experiment. The incident angle is set at the Bragg angle of the first diffraction ring of MHP, 4.36° for 12.7 keV. Measurement at the Bragg angle helps avoid the cutoff of the diffraction ring from the missing wedge, and this relatively high incident angle (compared to grazing incidence) gives a smaller footprint of the beam and results in a sharper diffraction peak. The sample-detector distance is set at 300 mm as a tradeoff between Q-range and Q-resolution. The ultimate Q resolution in our experiment is limited by the pixel size of the detector, corresponding to approximately 0.003 $\mathrm{\AA}^{-1}$ in reciprocal space. Figure S9 shows a photo of the in-situ stage inside the vacuum chamber at beamline 12-ID.

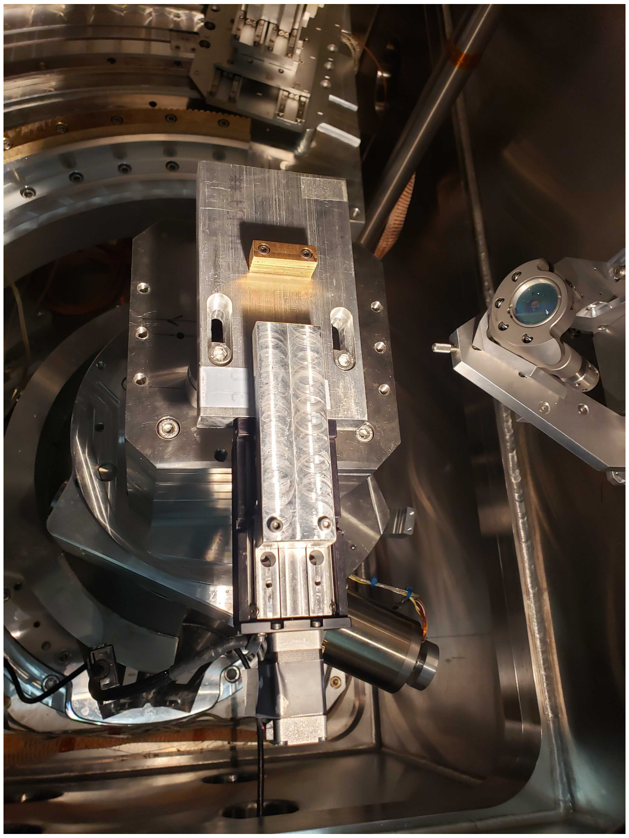

FIG. S9: In-situ bending stage at Soft Matter Interface (SMI) beamline, 12-ID, NSLS-II, Brookhaven National Lab.

VIII. REPEATABILITY

We performed measurements on several samples to evaluate repeatability. Figure S10 presents the applied versus measured strain for two I-2PACz samples with concave geometry, while Figure S11 shows the corresponding data for two Br-2PACz samples with convex geometry. The slopes of the curves are in good agreement, showing a consistent strain response. There are small differences in the absolute measured strain, which suggests that there is a small sample-to-sample variation in the initial strain state. This likely arises mainly from the intrinsic strain introduced during fabrication on flexible substrates. Note that the relative strain response is reproducible.

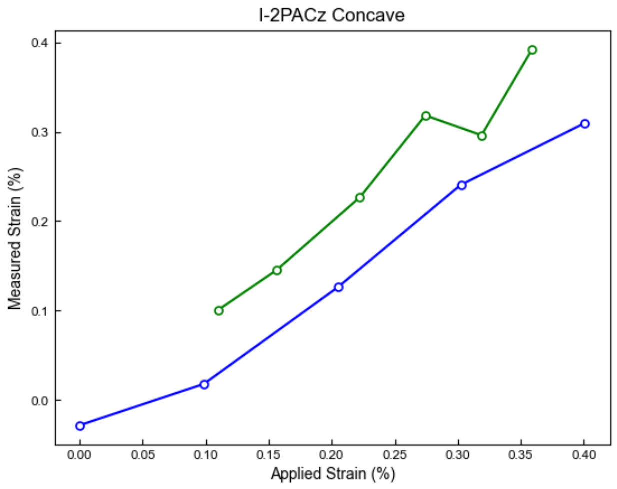

FIG. S10: Applied strain vs Measured strain for two I-2PACz samples under concave geometry.

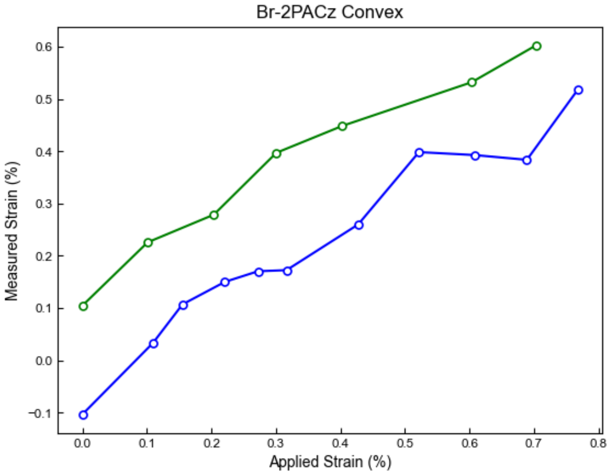

FIG. S11: Applied versus measured strain for two Br-2PACz samples under convex geometry, with strain defined as positive for convenience.

IX. ITO FILM BENDING TEST

In this work, we establish the relationship between interfacial slippage and the difference between applied and measured strain. However, the observed reduction in applied strain may also arise from cracking/wrinkling in the ITO thin film during bending. To rule out this possibility, we monitored the ITO surface after various bending conditions using Atomic Force Microscopy (AFM) and Scanning Electron Microscopy (SEM). Figures S12-S15 show the AFM images of the ITO side of the ITO-PET film at different bending radii. The samples are first bent in both convex and concave geometry (applied both tensile and compressive strain), and then measured in a flat state. Cracks/wrinkles in the ITO film are observed only under extreme bending conditions (Fig. S15), where the bending radius = 3.34 mm and the corresponding applied strain is 2.65%. An SEM of this film is shown in Figure S16. In our strain experiments, the applied strain was kept well below the level corresponding to the condition shown in Fig. S14. Therefore, the reduction in strain can be ruled out as originating from film cracking/wrinkling.

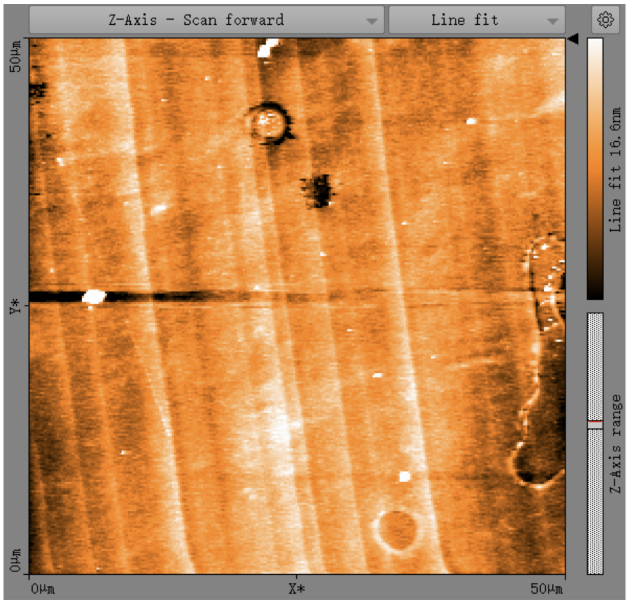

FIG. S12: AFM image of ITO-PET thin film with bending radius=12.64mm.

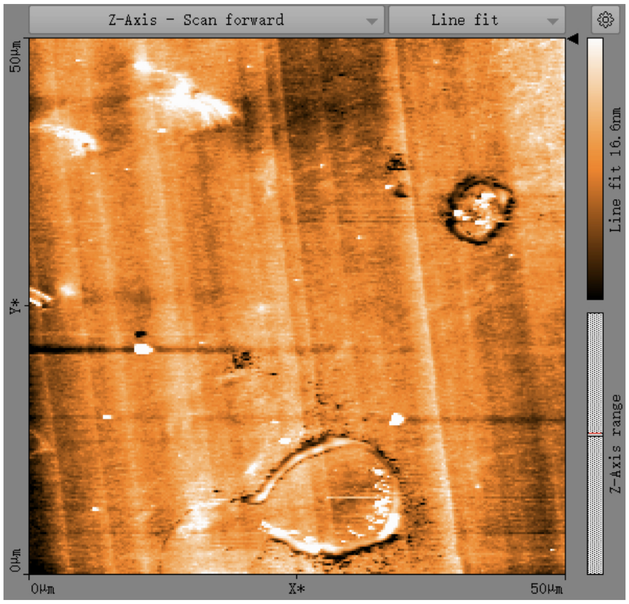

FIG. S13: AFM image of ITO-PET thin film with bending radius=7.14mm.

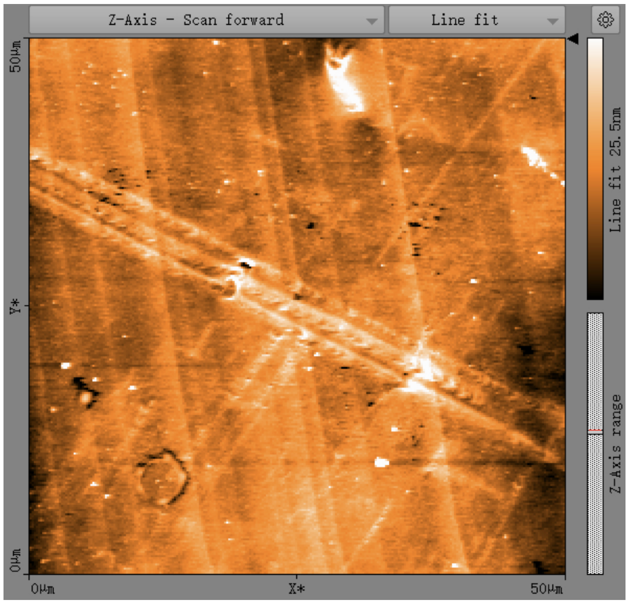

FIG. S14: AFM image of ITO-PET thin film with bending radius=4.78mm.

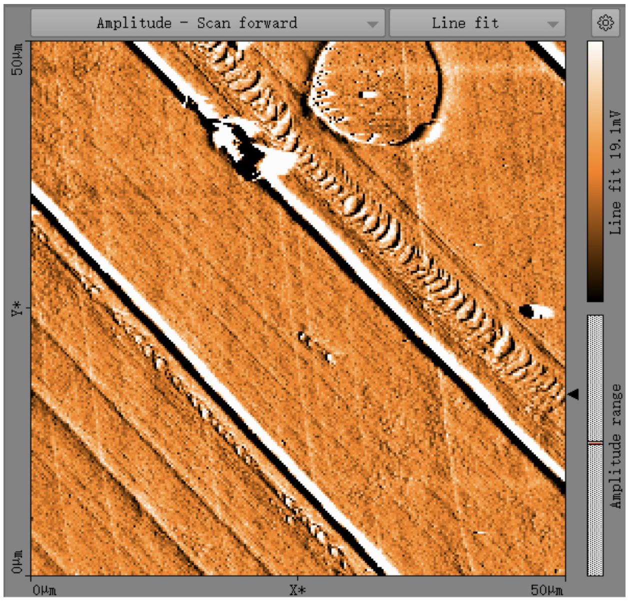

FIG. S15: AFM image of ITO-PET thin film with bending radius=3.34mm.

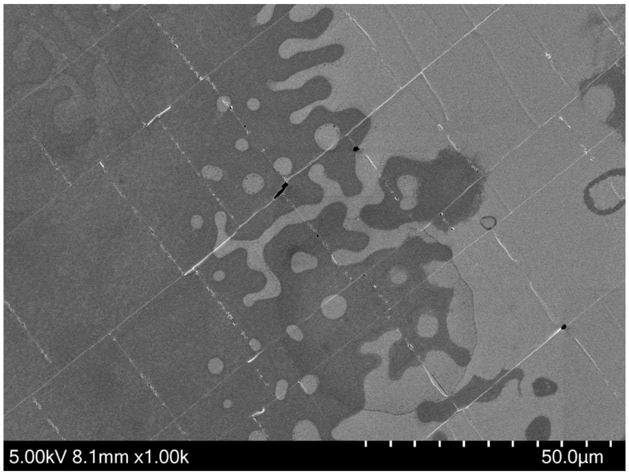

FIG. S16: SEM image of ITO-PET thin film with bending radius=3.34mm.
